# General Mass Property Measurement Equipment for Large-Sized Aircraft

**DOI:** 10.3390/s22103912

**Published:** 2022-05-21

**Authors:** Xiaolin Zhang, Hang Yu, Wenyan Tang, Jun Wang

**Affiliations:** School of Instrumentation Science and Engineering, Harbin Institute of Technology, Harbin 150001, China; hang_yu@hit.edu.cn (H.Y.); tangwy@hit.edu.cn (W.T.); wang_jun@hit.edu.cn (J.W.)

**Keywords:** mass property, integration measurement, load cell, mechanical structure, modeling

## Abstract

The accurate measurement of aircraft mass properties, such as the mass, centroid, and moment of inertia (MOI), plays a key role in the precise control of aircraft. In order to obtain high-precision information on the parameters of the mass, centroid, and MOI of an aircraft using a single instrument, an integrated mass property measurement system was developed in this study by analyzing and comparing the latest technologies, especially the function-switching device, which switches the measurement states between the center of mass and the MOI. The purpose of mass property measurement was achieved through single clamping. In addition, the system has strong versatility and expansion and can be used with different tooling or adapter rings to measure the mass properties of aircraft with different shapes. In this paper, the main mechanical structure of the measurement system, the measurement method of relevant mass parameters, and the solution method of the transformation matrix are introduced, and the standard parts and the aircraft were verified experimentally. The test results showed that the mass measurement accuracy was 0.03%, the centroid measurement error was within ±0.2 mm, and the measurement accuracy of the MOI was within 0.2%, all of which meet the high-precision measurement requirements for the mass properties.

## 1. Introduction

With the rapid development of aerospace technology in recent years, there have been unprecedented breakthroughs in the size and shape of aircraft. This is particularly important for the accurate measurement of mass properties, such as the mass, centroid, and MOI, which affect the precise control of aircraft. Mass is a measure of the inertia of an object, and the centroid is the geometric point of equivalent mass distribution. The measurement deviation of the centroid affects the dynamics modeling of the aircraft and the accuracy of the scalar equation of kinematics, which ultimately affects the flight trajectory of the aircraft [1,2,3,4,5]. The MOI is the measurement of the rotational inertia of a rigid body and plays a key role in the attitude control of aircraft in the air [6]. It can be said that the accurate acquisition of mass properties of aircraft, such as the mass, centroid, and MOI, is the premise of precise control of the aircraft.

In practical applications, it is usually necessary to measure multiple mass properties of the same product under test. The general method is to measure the centroid and MOI using a mass center measurement device and an MOI measurement device, respectively, for different measured parameters [2,7]. The parameters of the mass and centroid can be measured by inertial measurement, multi-point weighing, a compound pendulum or a three-wire pendulum, and other experimental testing methods. Linder investigated a model of a ship’s roll dynamics along with measurements from an inertial measurement unit [8]. Bacaro developed a device for measuring the CG location of satellites by finite element modeling and analysis (FEA) and multi-body modeling and simulation (MBS) [9]. Fabbri, LeClair, and Lemaire measured the center of gravity of narrow-track agricultural tractors, helmet systems, and patient wheelchairs according to the principle of torque balance using a static method [10,11,12]. Tang improved the pendulum method for the determination of the center of gravity for irregularly shaped bodies [13]. The MOI can also be measured by the hanging line torsion pendulum method or the torsion pendulum method. Previati measured the MOI of rigid bodies by means of multi-filar pendulums [14]. Furthermore, Liu proposed a method for measuring the inertia properties of a rigid body using a 3-URU parallel mechanism [15]. Nripen calculated the MOI of an aerospace vehicle by measuring the frequency of free torsional oscillation [16]. Bogdanov also designed a device that provided the MOI for an item of cylindrical form using the torsion pendulum method [17]. Brancati identified inertia parameters on the basis of the rigid-body complete motion equation and least-squares optimization [18]. Hou presented an improved approach for using a trifilar pendulum to identify parameters of oddly shaped bodies [19]. These measurement methods have been developed over a long period; they can achieve considerable accuracy when the measured part is of moderate size and regular shape, and they meet general measurement needs [11,14,18]. However, this method of separate measurement in the actual operation process involves the need for multiple clamping, which leads to more loading procedures, low efficiency, and the introduction of large positioning errors and other problems. If the test objects are small or medium-sized warheads or part of large-sized aircraft, the measurement system has a simple structure, and its measurement accuracy can be guaranteed even if the above factors are ignored [11,15]. However, with the increase in size, the diversification of shape, and the integration of the structure of aircraft, any small input error causes the error to be magnified several times in the output of measurement results, which makes it difficult to further improve the measurement accuracy of mass properties of large-sized aircraft. At the same time, in practical applications, for the measurement requirements of different types of aircraft, different test systems are usually redesigned to meet the requirements of installation, which causes a great waste of resources [5,6].

In order to solve the above problems, it is necessary to identify a strong, integrated design of mass property testing equipment that can reduce the positioning error introduced by multiple clamping to the maximum extent and improve the universality of the system. Wang C adopted a combination of the multi-line pendulum method and the hanging line torsion pendulum method to achieve multi-mass property measurement. However, this method has great limitations regarding the size of the tested product and is not suitable for the measurement of large-sized aircraft. By combining the multi-point weighing method and torsion pendulum method, Wang developed integrated mass property testing equipment suitable for large-sized aircraft [6]. However, the equipment achieved the switch in function of measurement of the centroid and MOI by moving the centroid measurement system up and down. This measurement method affects the relative position of the weighing sensor and the measured product and has a great impact on the accuracy of the centroid measurement.

In view of the shortcomings of mass property measurement technology of large-sized aircraft mentioned above, the following research was carried out in this study.

First, a mass property measurement system with a function-switching device that can shift measurement states between different mass properties (center of mass and MOI) of the aircraft flexibility was proposed. Another advantage of the device is that it can keep the relative position of the weighing sensor and the product under test unchanged under the condition that the position of the sensor is fixed, ensuring the accuracy of the measurement of mass properties. Second, this study designed and developed a multi-station switching device that provides a number of extensible interfaces. During the measurement process, the multi-station switching device matches the tooling or adapter ring for different objects being tested to achieve the clamping of different products. Because of the function of positioning in any quadrant position combined with the turning function provided by the tooling itself, unnecessary lifting and repeated installation links in the measurement process are reduced. Third, in order to omit high-precision coordinate measuring equipment, such as laser trackers, in actual measurement, a coordinate conversion idea is provided. A concept of a transition coordinate system was proposed. The measurement results can be converted into the coordinate system of the measured target without a laser tracker, which reduces measurement difficulty and saves time, after the initial calibration.

This paper introduces the basic principles of mass, centroid, and MOI measurement, and then describes the design principles of important mechanical structures in the integrated mass property measurement equipment. According to the structure of the measuring equipment, different coordinate systems were defined, and the coordinate transformation methods are given. Finally, the reliability of the equipment was verified by testing the standard parts and large-sized aircraft.

## 2. Materials and Methods

### 2.1. Theoretical Basis

#### 2.1.1. Principle for Obtaining Mass and CG

**Figure 1:**
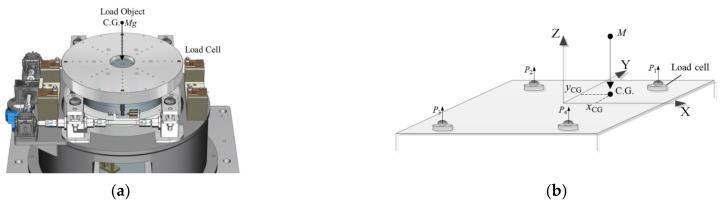
(**a**) The effect picture of the mass centroid measurement mechanism. (**b**) The schematic diagram of the mass centroid measurement.

As shown in Figure 1, the measurement system consists of four weighing sensors. In order to avoid the risk of overturning the measuring table when measuring large-sized aircraft, four-point weighing was adopted in the mass centroid measurement mechanism instead of the traditional three-point weighing.

Before measurement, the four weighing sensors are adjusted to the same height through the leveling mechanism of the equipment itself. First, the output values of the four sensors in the no-load state are recorded as *P*_11_, *P*_21_, *P*_31_, and *P*_41_, respectively, to obtain the equipment tooling mass Mg, namely [9]:

(1) $$M_{g}=P_{11}+P_{21}+P_{31}+P_{41}$$

**Figure 2:**
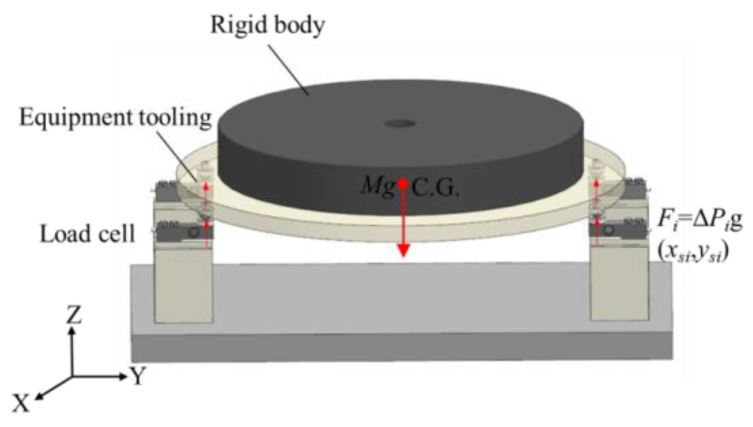
Measurement schematic diagram of mass centroid coordinates.

The test object is loaded as shown in Figure 2. The readings *P*_12_, *P*_22_, *P*_32_, and *P*_42_ of the four weighing sensors are summed again to obtain the total mass *M_z_* of the equipment tooling and the product, namely:

(2) $$M_{z}=P_{12}+P_{22}+P_{32}+P_{42}$$

The mass *M_c_* of the test object is the difference between the two measurement results:

(3) $$M_{c}=M_{z}-M_{g}$$

Furthermore, the 2D coordinates of the centroid of the test object can be calculated according to the output and position of the sensor using the principle of moment balance, and the coordinate system of the coordinates is the same as the coordinate system of the sensor coordinates involved in the calculation:

(4) $$x=\frac{\Delta P_{s1}x_{s1}+\Delta P_{s2}x_{s2}+\Delta P_{s3}x_{s3}+\Delta P_{s4}x_{s4}}{\Delta P_{s1}+\Delta P_{s2}+\Delta P_{s3}+\Delta P_{s4}}\;y=\frac{\Delta P_{s1}y_{s1}+\Delta P_{s2}y_{s2}+\Delta P_{s3}y_{s3}+\Delta P_{s4}y_{s4}}{\Delta P_{s1}+\Delta P_{s2}+\Delta P_{s3}+\Delta P_{s4}}$$

where *x_si_* and *y_si_* are the coordinates of the *i*th sensor in the coordinate system *xoy* plane, and Δ*P*_si_ is the difference between the measured values before and after the *i*th sensor is loaded with the test object.

Then, the coordinates are converted to the product coordinate system to obtain the centroid in the product coordinate system. Using this method, the centroid of the two directions can be obtained by one measurement. Similarly, after rotating the test object by 90° along its own *X*-axis and repeating the above measurement process, the 3D centroid coordinates of the test object can be obtained.

#### 2.1.2. Principle for Obtaining MOI

**Figure 3:**
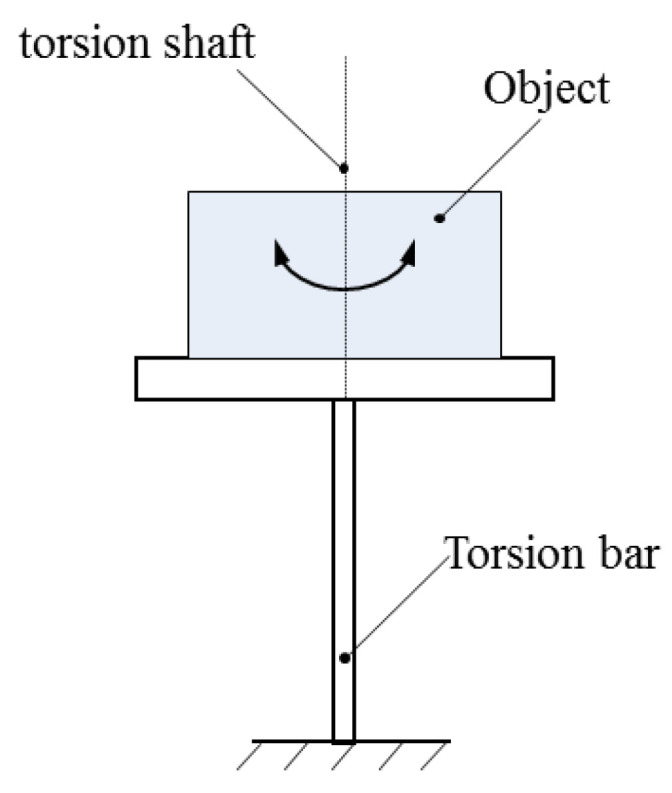
Schematic diagram of measuring MOI by torsional pendulum method.

As shown in Figure 3, the measurement system obtains the three-axis MOI (*X*-axis, *Y*-axis, and *Z*-axis) parameters of the test object through the torsion pendulum method. During the measurement, the test object is fixed on the horizontal torsion table; the measurement table and the test object are supported by the air bearing, and they are subjected to pure torsion pendulum vibration through the torsion bar. The MOI is calculated by measuring the torsional pendulum period. The MOI of the test object about the three coordinate axes of the centroid coordinate system is obtained according to the parallel axis theorem [6]:

(5) $$I=\frac{K}{{\left(2\pi \right)}^{2}}T^{2}$$

where *K* is the stiffness coefficient of the torsion bar determined by the material of the torsion bar, and *T* is the torsional pendulum period.

**Figure 4:**
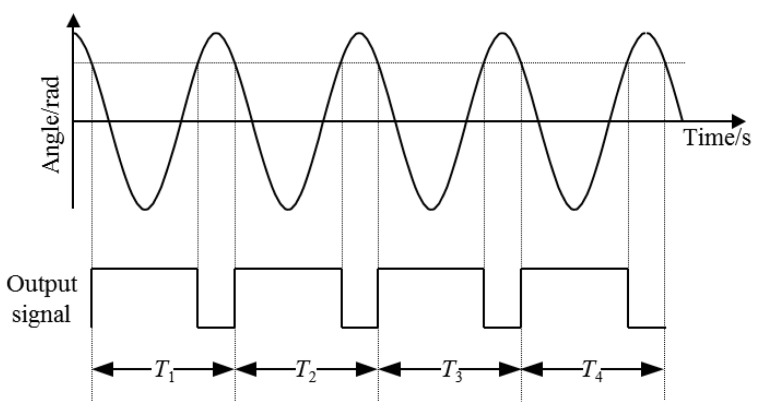
Schematic diagram of the measurement principle of the torsional pendulum period.

As shown in Figure 4, the period measurement adopts the switch timing method, which converts the torsion pendulum motion curve into a series of square wave signals with the switch, and the torsion pendulum period is the average value of the period of the square wave.

(6) $$T=\frac{T_{1}+T_{2}+\ldots +T_{n}}{n}$$

### 2.2. The Mechanical Structure

**Figure 5:**
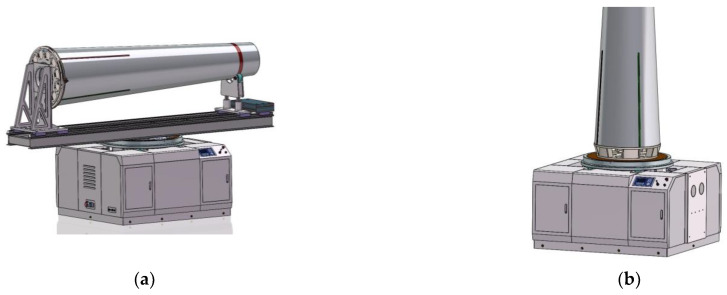
(**a**) Overall effect of the equipment—horizontal state; (**b**) overall effect of the equipment—vertical state.

In this section, the design of an integrated mass property measurement system based on the multi-point weighing and torsion pendulum method is described, along with the design and implementation of a new type of measurement function-switching mechanism, which can achieve the function of multi-parameter measurement and, at the same time, overcome the defects of current integration equipment. In the case of not using the coordinate measuring equipment outside the system, the relative position of the sensor and the test object is kept fixed to ensure the accuracy of mass property measurement. Furthermore, a multi-station switching device was developed to provide an expandable interface for the matching tooling of the test object with different dimensions and to provide the horizontal and vertical measurement functions for the test object, as shown in Figure 5. Through the function of quadrant positioning, unnecessary lifting and repeated installation links in the measurement process were reduced to the maximum extent, providing a new idea for the measurement of mass properties of large aircraft.

**Figure 6:**
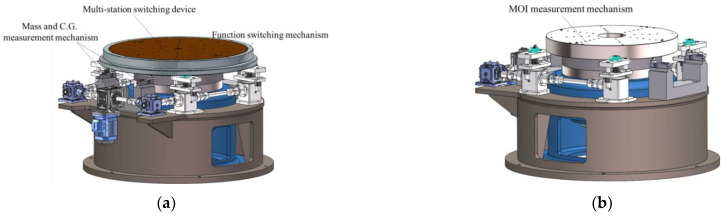
(**a**) The effect picture of mass property measuring table; (**b**) The effect picture of mass property measuring table without the multi-station switching device.

As shown in Figure 6, the measuring table for mass properties is the core part of the measurement system, which is composed of a multi-station switching device, a mass centroid measurement mechanism, a rotational inertia measurement mechanism, a function-switching mechanism, and other parts.

#### 2.2.1. Multi-Station Switching Device

**Figure 7:**
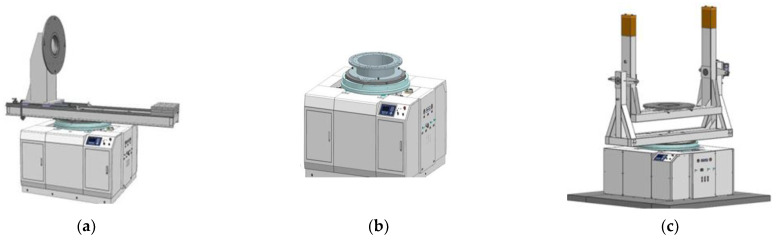
(**a**) Multi-station switching device and supporting tool—L-shaped bracket; (**b**) multi-station switching device and supporting tool—U-shaped bracket; (**c**) multi-station switching device and supporting tool—switching ring.

The functions of the multi-station switching device are as follows. First, it is connected to the measuring tooling and the product and plays the role of positioning and switching between the measuring tool and the measuring table. Second, it can locate and lock the product in the four quadrants of the measuring table, aiming to eliminate the systematic error of centroid measurement and improve the measurement accuracy. As shown in Figure 7, during the measurement process, the multi-station switching device achieves the clamping of different products by being compatible with the matching tooling (such as the L-shaped bracket and U-shaped bracket) or adapter rings of different test objects. The test object is not affected by factors such as the model, length, mass, or shape, which overcomes the limitation that most measuring instruments have of only being able to measure the test object of a single model in the previous mass property measurement.

**Figure 8:**
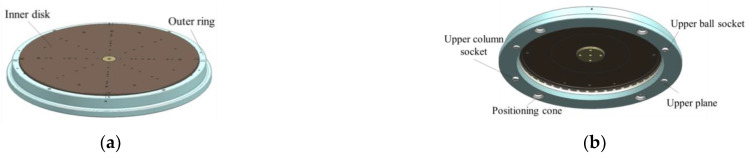
(**a**) The up view of the three-dimensional model of multi-station switching device. (**b**)The bottom view of the three-dimensional model of multi-station switching device.

The 3D model of the multi-station switching device is shown in Figure 8. The main body of the multi-station switching device is composed of an inner disk, a thrust ball bearing, and an outer ring. Multiple sets of concentric mounting screw holes, a central positioning hole, a quadrant positioning pin hole, and 8 positioning pin holes were designed on the inner disk to make the multi-station switching device compatible with the installation requirements of different tooling or products under test.

There are four positioning cones on the outer ring to coordinate with the positioning cones on the top of the lift to ensure accurate positioning of the multi-station switching device. The upper ball socket, the upper column socket, and the two upper planes are the steel ball positioning parts of the sensor components in the mass centroid measurement mechanism.

#### 2.2.2. Mass Centroid Measurement Mechanism

The function of the mass centroid measurement mechanism is to measure the mass and centroid of the test object by the multi-point weighing method. As shown in Figure 1a, it consists of four high-precision weighing sensors, an automatic centering assembly, a mounting pedestal of the weighing system, and other parts.

**Figure 9:**
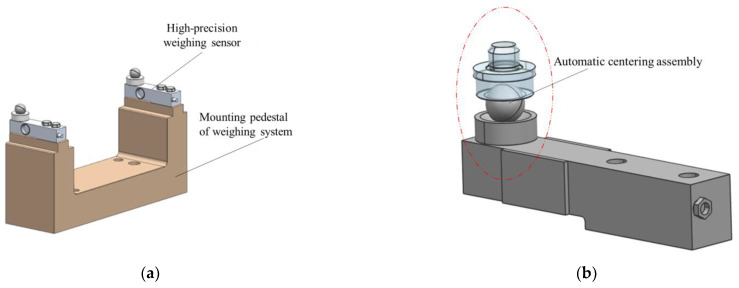
(**a**) Sensor measurement system model; (**b**) automatic centering assembly model.

As shown in Figure 9, during the measurement process, the object being measured and the tooling are installed and fixed on inner disk 2, and the position of the measurement table is adjusted to the measurement position of the mass centroid through the measurement function-switching device. At this time, the steel ball positioning member is pressed on the weighing sensor by the steel ball of the centering assembly [20]. The output information of the weighing sensor and its position information are recorded, and the centroid of mass is measured according to the principle of moment balance.

#### 2.2.3. MOI Measurement Mechanism

**Figure 10:**
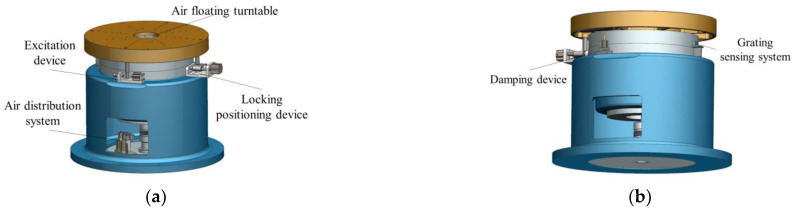
(a) Front view of the MOI measurement mechanism model; (**b**) Rear view of the MOI measurement mechanism model.

The function of the MOI measurement system is to complete the measurement of the rotational inertia of the test object through the torsion pendulum method. As shown in Figure 10, the MOI measurement system consists of an air floating turntable, an excitation device, a locking positioning device, a grating sensing system, a damping device, and an air distribution system.

Similar to the mass centroid measurement, it is necessary to install the test object and the tooling on the inner disk first and adjust the position of the measuring table to the measuring position of the MOI through the function-switching measuring device. During the measurement process, the initial excitation is applied to the air floating turntable through the air cylinder. Because of the stiffness of the torsion bar, the air floating table drives the tooling and the test object on it to complete the periodic torsion pendulum motion. By collecting the oscillation period signals obtained by the grating sensor system, the system calculates the MOI according to the torsional pendulum period.

#### 2.2.4. Function-Switching Mechanism

**Figure 11:**
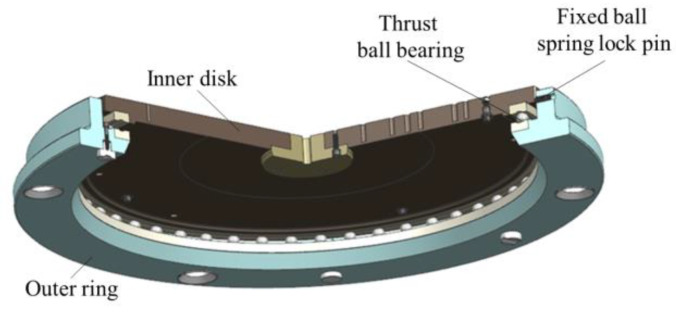
Model of function-switching mechanism.

The mass property measuring table can achieve function switching between the mass centroid parameter measurement and the MOI parameter measurement through the function-switching mechanism. This gives the measurement system the advantage of one-time clamping and measuring multiple physical quantities, which improves the measurement efficiency and makes the system structure more compact. As shown in Figure 11, similar to the multi-station switching device, the function-switching device includes an outer ring, an inner disk, and a thrust ball bearing. The inner disk and the thrust ball bearing are matched and connected, and the outer ring is coaxially connected with the inner disk.

In the centroid measurement, rotating the inner disk can cause the product on it to rotate. When turning to the fixed quadrant point, the inner disk and the test object are positioned, and the centroid of the product can be measured. When measuring the MOI, the elevator drives the multi-station switching device to drop, and when the inner disk and products fall onto the air floating turntable, the outer ring continues to drop and separate from the inner disk. Then, the outer ring and the thrust ball bearing are no longer in contact, and the fixed ball spring lock pin on the outer ring and the V-shaped groove of the inner disk are not connected. In other words, the MOI of the product can be measured.

**Figure 12:**
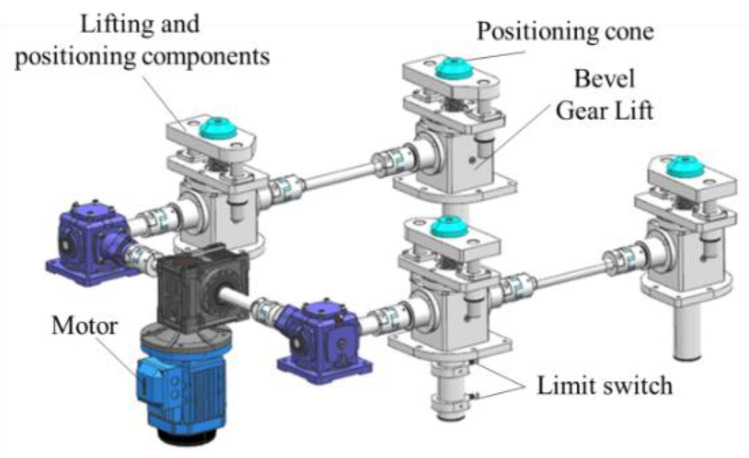
Model of lifting drive assembly.

As shown in Figure 12, the function-switching mechanism controls the lifting of the outer ring through the lifting transmission assembly and then achieves the switching of different measurement states, including the following three states.

- The non-measuring state

**Figure 13:**
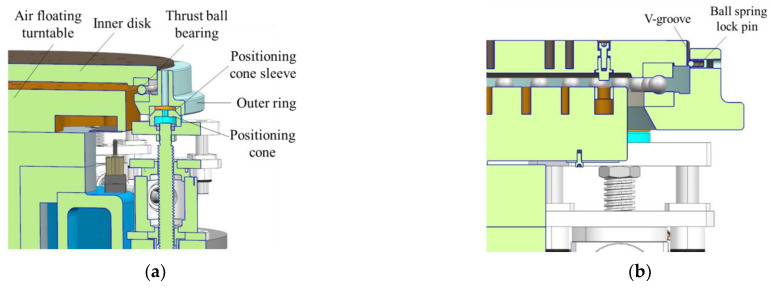
(**a**) The schematic of non-measuring state; (**b**) The detail view of non-measuring state.

As shown in Figure 13, the inner disc does not contact the air floating turntable, the inner disc falls on the outer ring through the thrust ball bearing, the ball spring lock pin fixed on the outer ring is just tangent to the V-groove of the inner disc, and the positioning cone of the lifting transmission system is supported on a positioning cone sleeve at the bottom of the outer ring.

- The mass centroid measurement state

**Figure 14:**
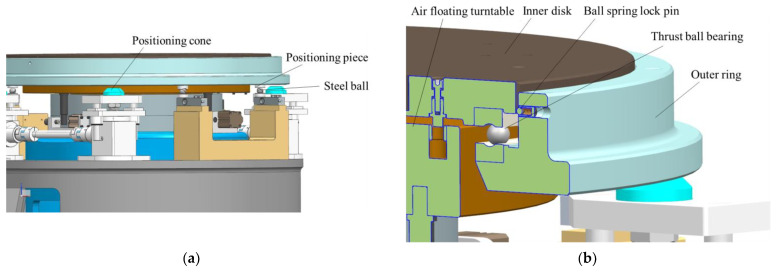
(**a**) The schematic of mass centroid measurement state; (**b**) The detail view of mass centroid measure state.

As shown in Figure 14, the inner disk does not contact the air floating turntable, the inner disk falls on the outer ring through the thrust ball bearing, and the ball spring lock pin fixed on the outer ring is just tangent to the inner disk groove. The positioning cone of the lifting transmission system does not contact the bottom positioning cone sleeve of the outer ring. The steel ball positioning piece fixed at the bottom of the outer ring is pressed against the sensor steel ball.

- The MOI measurement state

**Figure 15:**
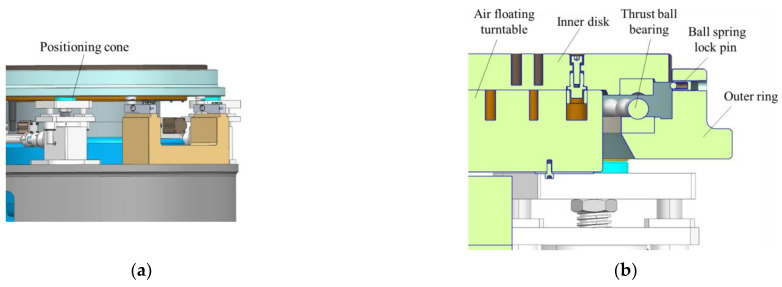
(**a**) The schematic of MOI measurement state; (**b**) The detail view of MOI measurement state.

As shown in Figure 15, the inner disk falls on the air floating turntable, the inner disk and the thrust ball bearing do not contact the outer ring, the positioning cone of the lifting transmission system supports the positioning cone sleeve at the bottom of the outer ring, and the steel ball is not placed on the sensor.

### 2.3. Coordinate System Setting and Coordinate Transformation

**Figure 16:**
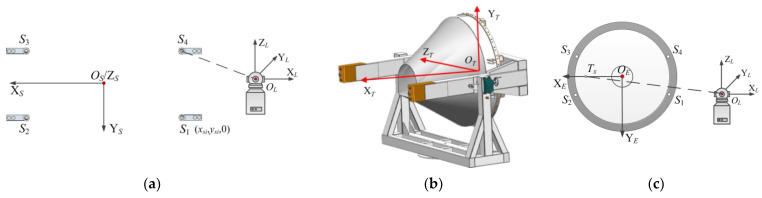
(**a**) The method of establishing SCS; (**b**) the method of establishing TCS; (**c**) the method of establishing ECS.

The sensor coordinate system (SCS), target coordinate system (TCS), and equipment coordinate system (ECS) are established sequentially according to the features of the measuring equipment. The SCS is determined during the commissioning of measuring equipment on the basis of the position of load cells. The CSC is established as shown in Figure 16a. The coordinates of the 4 sensor projection points can be expressed as *P_si_*(*x_si_*,*y_si_*,0), where *i* represents the number of the load cells. The TCS is set by the aircraft’s designer or user, and all measurements should be given on this basis. The general process of establishing the TCS of a common vehicle is shown in Figure 16b. Because of the complex mechanical construction of the measurement system and the obscuration of the instrument housing, the ECS is utilized as a transition coordinate system to alleviate the problem of not being able to establish the transformation matrix between SCS and TCS with a laser tracker. Figure 16c shows the process of building the coordinate system. The benefit is that the relative location between the load cells and the reference points remains constant, and the laser tracker does not need to be operated by a professional for each measurement.

Using the X and Y component values of the object’s center of mass as an example, the measurement results are shown in Equation (4), at which point the calculated coordinates of the center of mass are expressed in the SCS. It is necessary to transform the above data in order to obtain the centroid coordinates of the measured object in the TCS.

First, the laser tracker is used to measure the coordinates of key points in its own instrument coordinate system (LCS), the 4 sensor points can be expressed as $S_{L}{}_{i}(x_{Li},y_{Li},z_{Li})$, the centroid of the measured object can be expressed as $C_{L}(x_{C},y_{C},z_{C})$, and the coordinates of the origin O_E_, the positioning point T_X_ on *X*-axis, and the point T_Z_ on *Z*-axis on the ECS can be expressed as $O_{E}(x_{E},y_{E},z_{E})$, $T_{X}(x_{TX},y_{TX},z_{TX})$, and $T_{Z}(x_{TZ},y_{TZ},z_{TZ})$.

Then, the transformation matrix of the LCS and ECS is calculated; moreover, the rotation and the translation between coordinate systems are described by homogeneous coordinates. The unit direction vectors $\vec{e_{XE}}$, and $\vec{e_{ZE}}$ of the axes X_E_, Y_E_, and Z_E_ of the ECS under the LCS are noted as $m_{LE}=\left[\begin{array}{ccc} x_{XE} & y_{XE} & z_{XE} \end{array}\right]$, $n_{LE}=\left[\begin{array}{ccc} x_{YE} & y_{YE} & z_{YE} \end{array}\right]$, $p_{LE}=\left[\begin{array}{ccc} x_{ZE} & y_{ZE} & z_{ZE} \end{array}\right]$, and the coordinates of the origin O_E_ of the ECS in the LCS are $\left(x_{E},y_{E},z_{E}\right)$. The transformation matrix T_LE_ of the two coordinate systems is:

(7) $$T_{LE}=\left[\begin{array}{cccc} x_{XE} & x_{YE} & x_{ZE} & x_{E}\\ y_{XE} & y_{YE} & y_{ZE} & y_{E}\\ z_{XE} & z_{YE} & y_{ZE} & z_{E}\\ 0 & 0 & 0 & 1 \end{array}\right]$$

Therefore, in the ECS, the 4 supporting points of the sensor group can be described as $T_{LE}^{-1}{\left[x_{si}\;\;y_{si}\;\;z_{si}\right]}^{T}$, and the object’s centroid is $T_{LE}^{-1}{\left[x_{C}\;\;y_{C}\;\;z_{C}\right]}^{T}$.

Finally, after loading the tooling and the object, the position of the 4 supporting points in the ECS does not change. The transformation matrix TEO between the TCS and the ECS can be obtained according to the tooling with known dimensions. In the TCS, 4 sensor points can be expressed as $T_{EO}^{-1}T_{LE}^{-1}{\left[x_{si}\;\;y_{si}\;\;z_{si}\right]}^{T}$, and the centroid of the tested object can be expressed as $T_{EO}^{-1}T_{LE}^{-1}{\left[x_{C}\;\;y_{C}\;\;z_{C}\right]}^{T}$.

## 3. Results

### 3.1. Calibration Result

**Figure 17:**
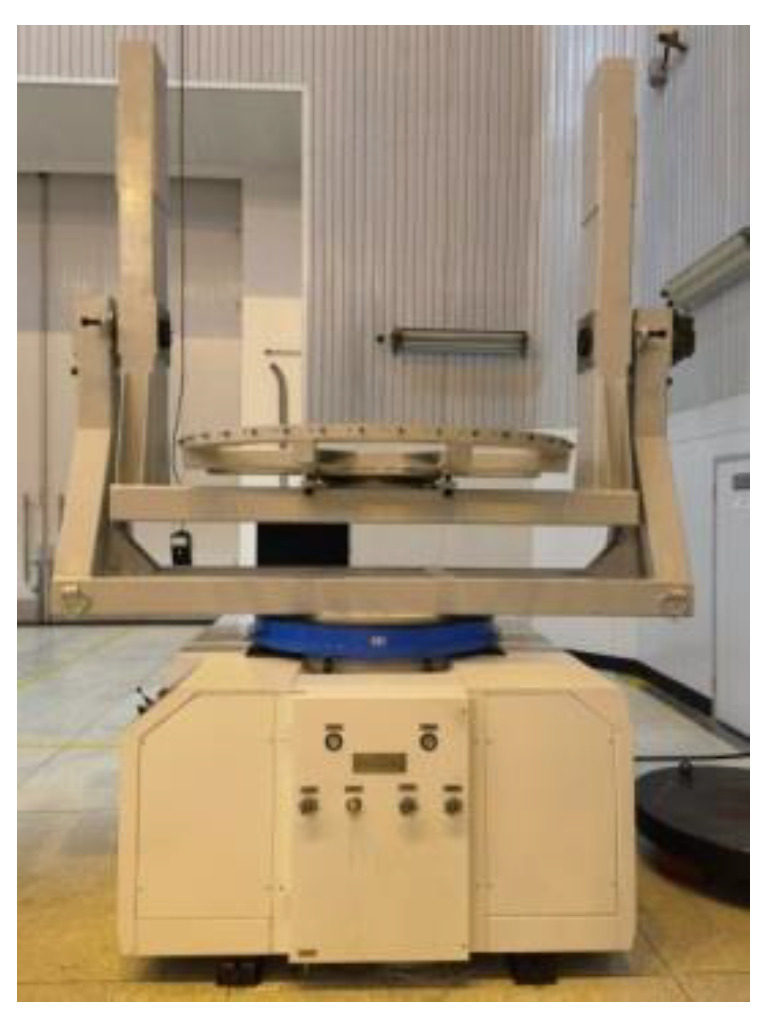
Physical picture of the measurement system.

As shown in Figure 17, the integrated mass property measurement equipment was developed for the features of large-sized aircraft and the measurement principle mentioned above, and the whole set of measurement processes could be completed by one hoisting. The parameters of the system are given in Table 1. The test item measurement accuracies are given in Table 2.

**Figure 18:**
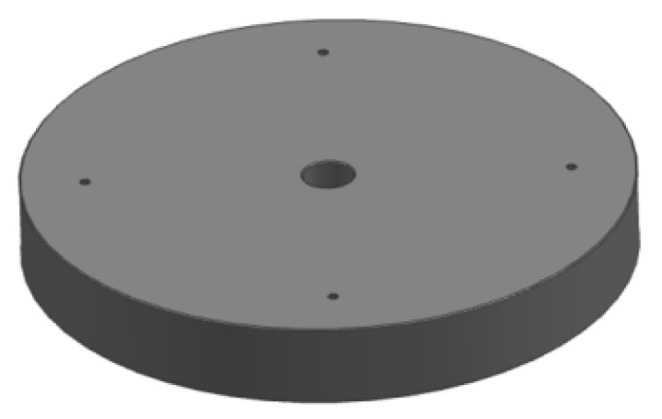
Three-dimensional model of standard parts.

Before measuring the aircraft, we carried out experiments with standard parts. The standard parts are round cake-shaped structures made of 45# high-quality carbon steel with a fixed size positioning hole in the center, convenient for installation and positioning with the multi-station switching device, as shown in Figure 18. The mass and center of gravity of the standard parts were calibrated by the metering mechanism. The calibration results showed that the mass was 883.495 kg, and the deviation between the center of gravity and the center of mass was within 0.05 mm, indicating that the center of gravity and the center of mass coincided.

**Figure 19:**
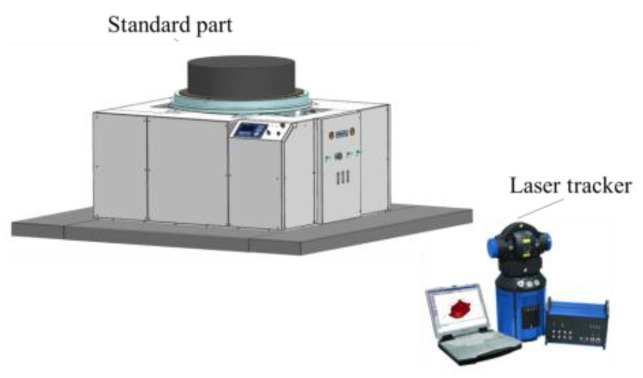
Standard sample mass centroid measurement diagram.

**Figure 20:**
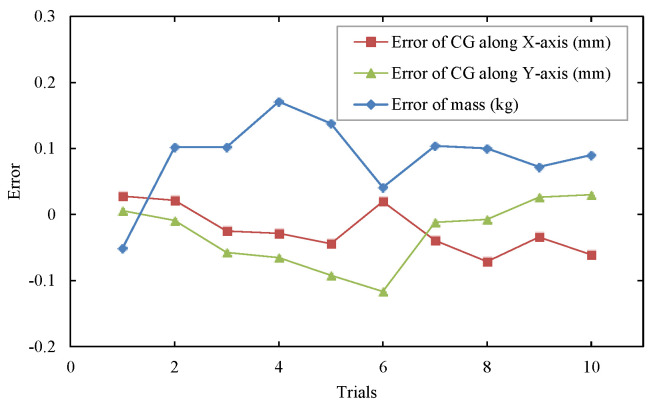
Standard sample mass centroid measurement error.

During the test, the standard parts were placed in ten different positions on the measuring table; the geometric center positions of the upper plane of the standard parts were measured by a laser tracker and compared with the measurement results. The measurement plan is shown in Figure 19. The result is shown in Figure 20.

It can be seen that the error (2σ) of the mass parameters of the integrated mass parameter measurement system was no more than 0.2 kg, and the mass measurement accuracy (2σ) reached 0.02%. The centroid measurement error uncertainty (2σ) was within 0.1 mm.

**Figure 21:**
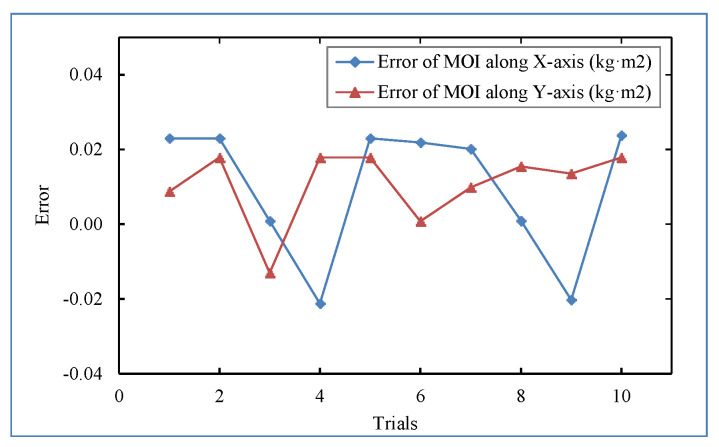
Standard sample MOI measurement error.

The MOI of the standard part was measured by the rotational inertia measurement mechanism. A standard disk with a mass of 883.495 kg, a radial MOI of 134.14 kg·m^2^, and an axial MOI of 71.260 kg·m^2^ was used as the standard part. A total of 10 measurements were completed. During the measurement, the standard disk was fixed in the same position on the measuring table, and all the measurements were completely collected. The measurement results are shown in Figure 21. The rotational inertia uncertainty (2σ) was not greater than 0.04 kg·m^2^, and the repeatability accuracy (2σ) was not greater than 0.2%.

From the above measurement results, it can be seen that the integrated mass property measurement equipment can accurately measure the mass centroid and MOI.

### 3.2. Example

According to the measurement methods and key points described in Section 2 and Section 3, the mass centroid and MOI of large-sized aircraft with a mass of about 250 kg were measured. Ten groups of experimental cycles were completed, and the results are shown in Table 3.

It can be calculated from Table 3 that the error (2σ) of the mass parameter was not greater than 0.1 kg, and the repeatability accuracy (2σ) of the mass parameter was not greater than 0.03%. The error uncertainty (2σ) of the axial CG was 0.16 mm, and the error uncertainty (2σ) of the radial CG was not greater than 0.1 mm. The repeatability accuracy (2σ) of the vertical MOI and the horizontal MOI were both within 0.1%, which meets the accuracy index proposed in Table 2.

Table 4 shows the average values of the ten test groups. By analyzing the 3D model of the test object, we obtain its mass properties (mass, centroid, and MOI), which are listed in Table 4. The comparison of these data shows that the integrated mass property measurement equipment introduced in this paper achieved a high degree of coincidence with the simulation results.

## 4. Discussion

At present, the mass centroid and MOI are widely used using the method of “separated measurement of different equipment”. In this study, through the sophisticated mechanical structure design combined with the high-precision measurement principle, a mass property measurement table was proposed and developed, which is a comprehensive and integrated measurement device integrated with mass, centroid, and MOI measurement functions. Comparing the two, the latter has significant advantages, such as a small equipment footprint, fewer product installations, high measurement accuracy, and strong compatibility, and the general mass property measurement equipment is also in line with future development trends.

The instrument is suitable for objects weighing up to 3000 kg, and the measurement results for large-sized aircraft demonstrate the effectiveness of the device. The test object is not limited to a single shape or size; it can also be extended to the test objects beyond aerospace.

## Figures and Tables

**Table 1:** Major dimensions of the systems.

Item	Dimensions
Height of each system	1052 mm
Length of each system	1900 mm
Width of each system	1680 mm
Accuracy of the load cells	0.02%
Distance measurement accuracy of laser tracker	10 μm ± 0.5 μm/m
Angle measurement accuracy of laser tracker	±15 μm + 6 μm/m

**Table 2:** Specification and design requirements of the systems.

Item	Dimensions
Test parameters	Mass; CG along axes X, Y, and Z; MOI along axes X, Y, and Z
Mass measurement range	100–3000 kg
Mass measurement accuracy	≤0.03%
Axial CG measurement accuracy	≤±0.5 mm
Radial CG measurement accuracy	≤±0.15 mm
Axial MOI measurement accuracy	≤±0.5%
Radial MOI measurement accuracy	≤±0.2%
Applicable product maximum diameter	No less than φ1650 mm

**Table 3:** Measurement results of mass properties.

Test No.	Coordinate of CG (mm)			Mass (kg)	Value of MOI (kg·m^2^)		
	X	Y	Z		X	Y	Z
1	505.958	28.120	−4.056	258.529	101.113	67.139	74.323
2	506.077	28.038	−4.073	258.632	101.113	67.148	74.411
3	505.913	28.022	−4.059	258.632	101.091	67.117	74.403
4	505.874	28.044	−4.079	258.641	101.069	67.148	74.355
5	505.924	28.012	−4.098	258.628	101.113	67.148	74.348
6	505.902	28.016	−4.090	258.621	101.112	67.131	74.347
7	506.007	28.022	−4.070	258.640	101.110	67.140	74.308
8	506.008	27.987	−4.042	258.680	101.091	67.145	74.394
9	506.101	28.001	−3.975	258.652	101.070	67.144	74.343
10	506.083	27.993	−4.046	258.670	101.114	67.148	74.407

**Table 4:** Comparison between measurement results and simulation results.

Test No.	Coordinate of CG (mm)			Mass (kg)	Value of MOI (kg·m^2^)		
	X	Y	Z		X	Y	Z
Experimental results	505.985	28.025	−4.059	258.633	101.100	67.141	74.364
Simulation results	505.990	28.010	−4.04	258.58	101.090	67.130	74.330
Difference	−0.005	0.015	−0.019	0.053	0.010	0.011	0.034
